# Lead Structures for Applications in Photodynamic Therapy. 6. Temoporfin Anti-Inflammatory Conjugates to Target the Tumor Microenvironment for *In Vitro* PDT

**DOI:** 10.1371/journal.pone.0125372

**Published:** 2015-05-19

**Authors:** Luke Rogers, Natalia N. Sergeeva, Edyta Paszko, Gisela M. F. Vaz, Mathias O. Senge

**Affiliations:** 1 School of Chemistry, SFI Tetrapyrrole Laboratory, Trinity Biomedical Sciences Institute, 152–160 Pearse Street, Trinity College Dublin, The University of Dublin, Dublin, 2, Ireland; 2 Medicinal Chemistry, Institute of Molecular Medicine, Trinity Centre for Health Sciences, Trinity College Dublin, St. James's Hospital, Dublin, 8, Ireland; University of Pécs Medical School, HUNGARY

## Abstract

Due to the ongoing development of clinical photodynamic therapy (PDT), the search continues for optimized photosensitizers that can overcome some of the side effects associated with this type of treatment modality. The main protagonists being: post-treatment photosensitivity, due to only limited cellular selectivity and post-treatment tumor regrowth, due to the up-regulation of pro-inflammatory agents within the tumor microenvironment. A photosensitizer that could overcome one or both of these drawbacks would be highly attractive to those engaged in clinical PDT. Certain non-steroidal anti-inflammatory drugs (NSAIDs) when used in combination with PDT have shown to increase the cytotoxicity of the treatment modality by targeting the tumor microenvironment. Temoporfin (*m*-THPC), the gold standard chlorin-based photosensitizer (PS) since its discovery in the 1980’s, has successfully been conjugated to non-steroidal anti-inflammatory compounds, in an attempt to address the issue of post-treatment tumor regrowth. Using a modified Steglich esterification reaction, a library of “iPorphyrins” was successfully synthesized and evaluated for their PDT efficacy.

## Introduction

Photodynamic therapy (PDT) is a still developing treatment modality for solid tumors of the head, neck, throat, peritoneal cavity, skin as well as non-oncologic maladies such as psoriasis or age-related macular degeneration [1,2]. The treatment involves the use of a three component system comprising of a photosensitizer, light of specific wavelength and singlet oxygen. The light causes the photochemical generation of cytotoxic oxygen species, among them most prominent ^1^O_2_ by the photosensitizer within the target tissue. These reactive oxygen species contribute to cell death through both apoptotic and necrotic pathways [3]. PDT-mediated oxidative stress has shown to include an array of immunologic responses and signal transduction pathways whose impact on the efficacy of the treatment is still relatively unknown [4].

PDT as a treatment is continually evolving, with improvements in photosensitizer efficacy, light source and dosimetry being made constantly [5,6]. There are a number of new photosensitizers under investigation that have displayed improved clearance rates, targeting properties or photophysical profile [7,8]. However, research and advancements in areas such as post-treatment photosensitivity and tumor regrowth still need addressing [4]. Note, that the *in vivo* toxicity of these PS is dependent on three factors: drug concentration and action, light and oxygen concentration. Thus any pharmacological investigation is more complex than that of ‘classic’ chemotherapeutic drugs. On the other hand the multimodal mode of action through reactive oxygen species mostly prevents resistance to develop.

PDT can induce inflammation and hypoxia that leads to the increased expression of pro-survival and angiogenic molecules such as vascular endothelial growth factor (VEGF), survivin and matrix metalloproteinases (MMPs) [9,10]. This up-regulation of pro-inflammatory molecules within the tumor microenvironment can decrease the efficacy of the treatment due to the occurrence of tumor regrowth [4,11].

Researchers have found that the prostaglandin-endoperoxide synthase 2 (COX-2) gene is consistently over-expressed after a course of PDT and this result coincides with previous work that demonstrated that PDT stimulates the release of prostaglandin E2 (PGE_2_) from macrophages and radiation induced fibrosarcoma (RIF) cells grown in culture [12]. This endogenous prostaglandin (PG) release in mice exhibiting a systemic shock reaction after localized PDT could be attenuated with NSAIDs [13,14]. Co-administration of celecoxib with PDT improved the long-term tumoricidal activity whilst preserving normal tissue photosensitization [11,15]. Unfortunately, the administration of COX-2 inhibitors can cause a number of side effects, which include cardiovascular or gastrointestinal injuries [16].

Studies indicate that the anti-tumor actions of coxibs such as celecoxib are independent of COX-2 mediated mechanisms. It has been shown that a celecoxib analogue, 2,5-dimethyl celecoxib (DMC), displays cytotoxic properties comparable to celecoxib but without the COX-2 inhibitory activity. Gomer and coworkers were able to demonstrate the efficacy of DMC using a C3H/BA murine mammary carcinoma model. Results showed that DMC increased both the cytotoxicity and apoptosis in BA cells exposed to PDT treatment along with the induced expression of the pro-survival protein survivin [17]. PGE_2_ levels remained unchanged despite the enhanced *in vivo* tumoricidal responsiveness to PDT, which means that DMC improved the efficacy of the treatment by causing an increase in apoptosis and anti-tumor activity.

Whilst there is strong evidence for improved efficacy with the co-administration of an anti-inflammatory drug, to our knowledge, no research has been conducted on the administration of a photosensitizer-anti-inflammatory agent conjugate [12,17]. We theorize that a conjugate would undergo biological cleavage to give the PS and anti-inflammatory drug, which would accommodate localized application of the anti-inflammatory agent and therefore an increased therapeutic response. We here present initial data on the synthesis and biological evaluation of such compounds.

## Results and Discussion

### Synthesis

**Fig 1:**
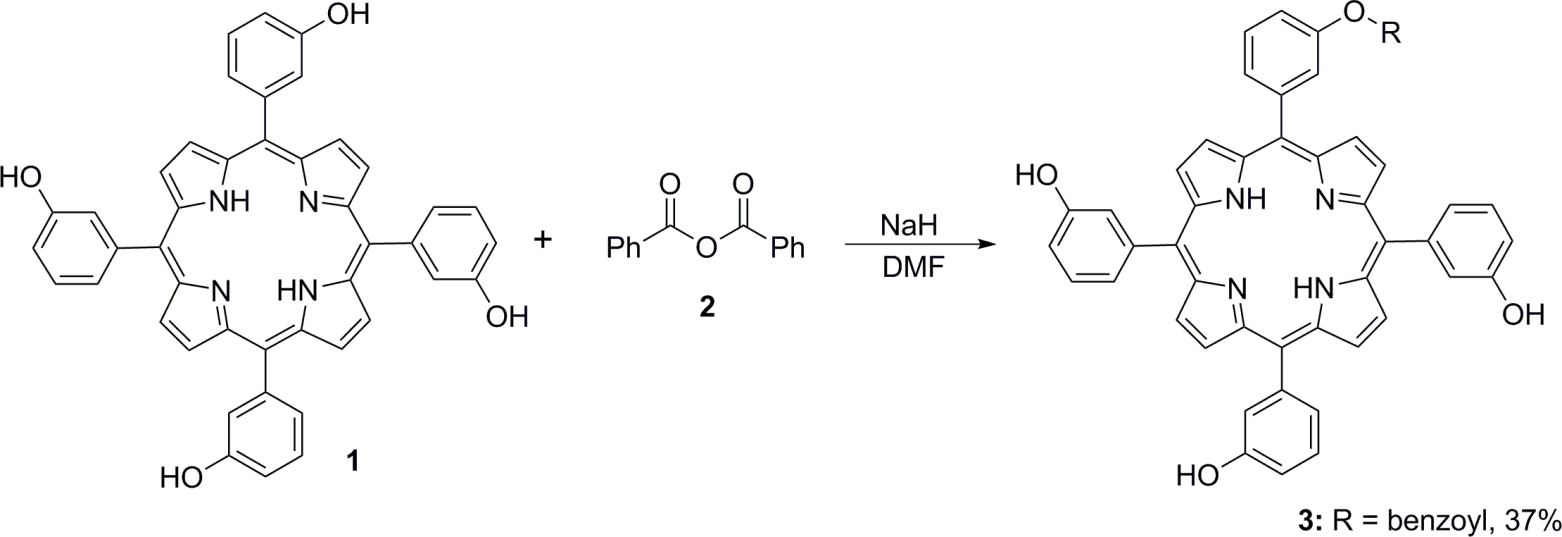
Controlled functionalization of the *m*-THPP photosensitizer scaffold using benzoic anhydride.

Work on this project began with the continuation of studies conducted previously on the controlled functionalization of porphyrin-based photosensitizers through simple nucleophilic substitution, esterification reactions or metal-catalyzed cross-coupling reactions. By using a simple aromatic anhydride **2**, it was possible to selectively functionalize the porphyrin periphery to the desired degree of substitution by monitoring the reaction time and equivalents used (Fig 1) [18].

In an attempt to avoid any potential cardiovascular pitfalls caused by coxib based anti-inflammatories, a library of NSAIDs was chosen for the synthesis of these iPorphyrin conjugates [16]. As the carboxylic acid functionality is present in most NSAIDs, and following on from our work on the functionalization of known PSs, 5,10,15,20-tetrakis(3-hydroxyphenyl)porphyrin (*m*-THPP) and 5,10,15,20-tetrakis(3-hydroxyphenyl)chlorin (*m*-THPC), it seemed a logical step to attempt the synthesis of these conjugates through esterification reactions. The ester functionality is a bond that readily undergoes enzymatic cleavage and can be easily installed into molecules. There are also wide arrays of NSAIDs that are commercially available and therefore provide a large scope for variation for a library of conjugates.

We wished to use this newly developed methodology to functionalize Temoporfin (*m*-THPC) [19,20,21], the gold standard of *meso*-substituted chlorin-based photosensitizers with known molecules used to treat inflammation. Fenamic acid serves as a parent structure to a number of different NSAIDs, *e*.*g*., tolfenamic acid and flufenamic acid. However, for an initial screen of the utility of this approach we wished to optimize our synthetic pathway with a cheap and readily available synthetic precursor, *i*.*e*. 5-hydroxyanthranilic acid, **4**. This molecule contains many of the functional groups present in fenamic acid and other NSAIDs and provides an initial increase in complexity from the simple anhydride used in previous work [18].

The initial screen of conditions began with the use of a simple Fischer esterification reaction to yield the chlorin ester product. A catalytic amount of H_2_SO_4_ was used with dry CH_2_Cl_2_ and left to heat at reflux overnight. This reaction proved unsuccessful, even at increased temperatures, and it seems that the chlorin macrocycle may be interfering with the electronics of the phenol group by reducing the nucleophilicity of the hydroxy group and therefore inhibiting the reaction with the carboxylic acid.

A Steglich reaction [22] using *N*,*N'*-dicyclohexylcarbodiimide (DCC) and 4-dimethylaminopyridine (DMAP) as coupling partners was attempted in DMF at room temperature. Initial results from this reaction appeared promising, however, the yield for the reaction was low and the presence of an insoluble *N*-acyl urea made purification of the product problematic.

A suitable substitute for DCC is 1-ethyl-3-(3-dimethylaminopropyl)carbodiimide hydrochloride (EDC), a preferred alternate cross-coupling agent due to its solubility in water [23]. This was used in the presence of a catalyst, DMAP, in DMF and at room temperature with no observable esterification occurring. The reaction was reattempted at elevated temperatures and no reaction product was observed by TLC and NMR.

Hydroxybenzotriazole (HOBt) may also be used in conjunction with EDC as a carboxy-activating agent [24,25]. It reacts with the *O*-acylurea formed from the reaction between the carboxylic acid group of the NSAID and the EDC, to form an activated ester, which can be isolated or used *in situ* [26,27]. Generation of this activated ester and subsequent addition of the chlorin photosensitizer allowed for the formation of the ester conjugates **8a** and **8b**. By monitoring the reaction time and equivalents, one is able to maximize the amount of mono-functionalized chlorin in the reaction mixture in comparison to the higher order derivatives. Thin-Layer chromatography showed a distinctive degree of substitution pattern, with the tetra-functionalized derivative having the highest *R*_*f*_ and the unsubstituted chlorin having the lowest.

**Fig 2:**
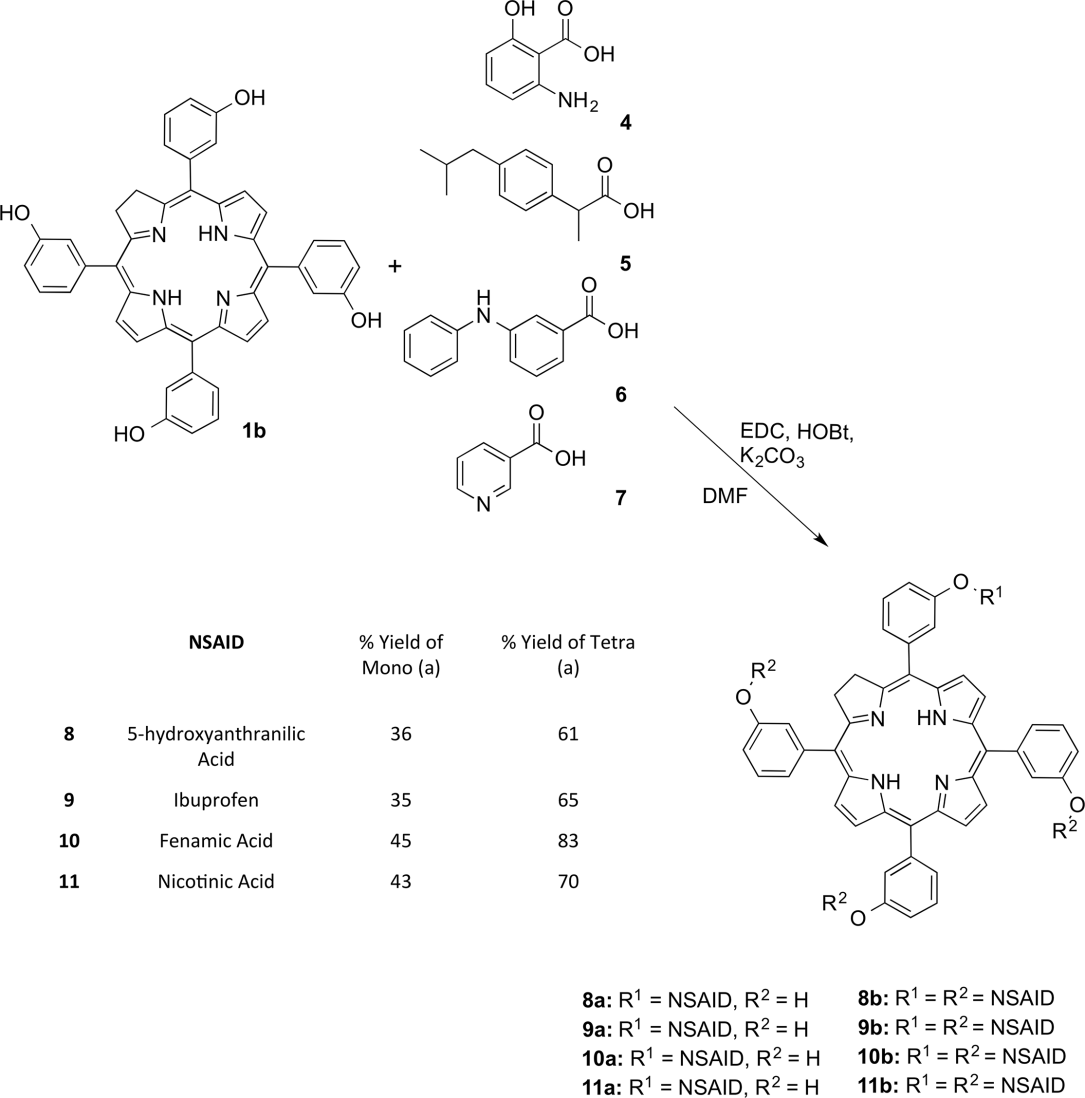
Modified Steglich reaction with a variety of NSAIDs to afford novel chlorin-NSAID photosensitizers. *Only one of the possible isomers is shown.

Following on from the success of these reaction conditions, the scope of the synthesis was investigated by using a variety of NSAIDs to create a small library of conjugates. Ibuprofen, (*S*)-2-(4-(2-methylpropyl)phenyl)propanoic acid **5** and fenamic acid, *i*.*e*. 2-(phenylamino)benzoic acid **6** were chosen as suitable initial candidates for the screen due to similarities in structure to anthranilic acid and also their prolific record as agents used in the treatment of numerous inflammatory diseases [28,29,30]. Both NSAIDs readily conjugated to the chlorin scaffold in a controlled and predictable fashion and altering the reaction time and equivalents of reagents used could maximize the degree of functionalization and achieve conjugates **9a**, **9b**, **10a** and **10b** in good yields as per the Fig 2.

The final section of this library of conjugates was completed with the introduction of Nicotinic acid **7** onto the chlorin periphery. Nicotinic acid, whilst traditionally clinically implemented to influence cholesterol levels, has been shown to have quite promising anti-inflammatory properties in cases of atherosclerosis [31,32]. Unfortunately, nicotinic acid did not readily undergo conjugation with the chlorin photosensitizer under these optimized conditions. However, after investigation into a number of different bases and modified procedures, it was found that pyridine and EDC worked to yield the mono- (**11a**) and tetrafunctionalized (**11b**) derivatives in yields comparable to the previous conjugates.

This library has shown that *m*-THPC is tolerable of standard reaction conditions, work-ups and chromatography without subsequent oxidation to the parent porphyrin derivative; evidence that contradicts historical perceptions of this molecule. NMR assignment of the mono-functionalized compounds proved extremely difficult due to the presence of a statistical mixture of the possible regiochemical isomers. These compounds were prepared for comparative purposes to gain some initial insight in the influence of the substituent pattern and amphiphilicity on the uptake. These regioisomers are formed because of the reduced double bond located on the pyrrole A ring and they cannot be resolved using standard chromatographic procedures. This mixture is consistent with literature reports of similar systems, however, where possible, the *β* and inner pyrrolic protons were used as diagnostic peaks [33,34,35]. Thus, high-resolution mass spectrometry (HRMS) analysis acted as our main analytical tool for confirmation of product formation and NMR was used for the assignment of the dominant regioisomer being presented in the experimental section.

### Singlet Oxygen Studies

For any molecule to be considered a potential clinical PS, they must possess a high quantum yield of ^1^O_2_. To evaluate the ^1^O_2_ production capability of our library of conjugates, compounds were subjected to a crude assay in order to estimate their ^1^O_2_ generation [36]. Their production of ^1^O_2_ was determined through the monitoring of the change in the absorption at 410 nm of the dye 1,3-diphenylisobenzofuran (DPBF) in solution. DPBF has an absorbance maximum at 410 nm and readily undergoes a ring opening reaction in the presence of ^1^O_2._ One can monitor the reduction of this band over time and graph an estimate of ^1^O_2_ production. Compounds **9b** and **10a** were taken as representative samples of the library and screened for their singlet oxygen production capabilities. The results obtained for compounds **9b** and **10a** from this assay were then compared to results obtained with the known ^1^O_2_ producer *m*-THPC to give a relative indication of their ^1^O_2_ producing potential.

**Fig 3:**
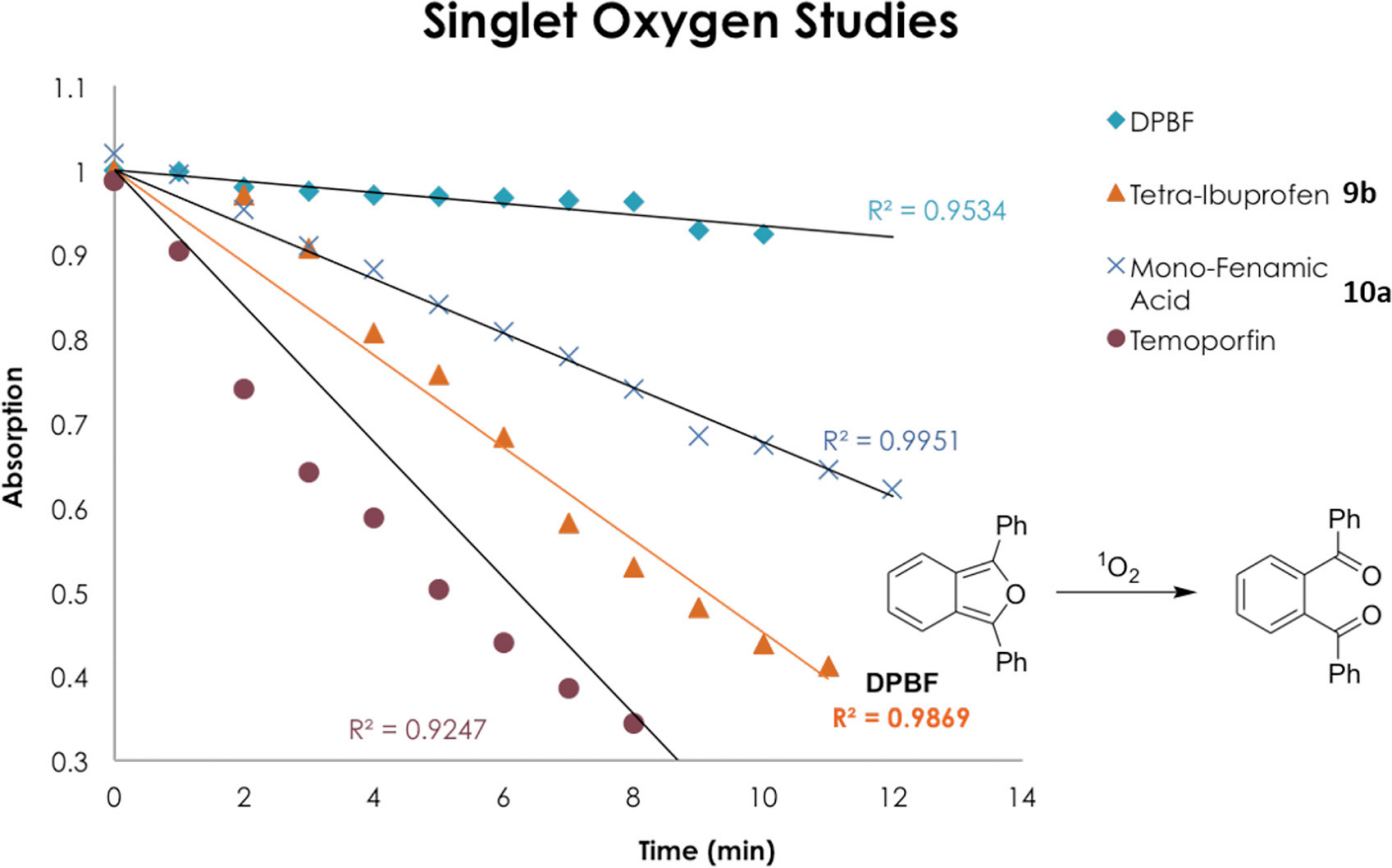
Graph displaying the reduction in absorbance of DPBF at 410 nm over time due to the production of ^1^O_2_ by 9b and 10a.

Compounds **9b** and **10a** were dissolved in an aerated solution of DPBF in DMF and irradiated with white light and the intensity of the DPBF’s absorption band was monitored over time using UV-vis spectroscopy. Both compounds showed good production of ^1^O_2_, as per Fig 3, however not to the same extent as *m*-THPC.

### Biological Testing

**Fig 4:**
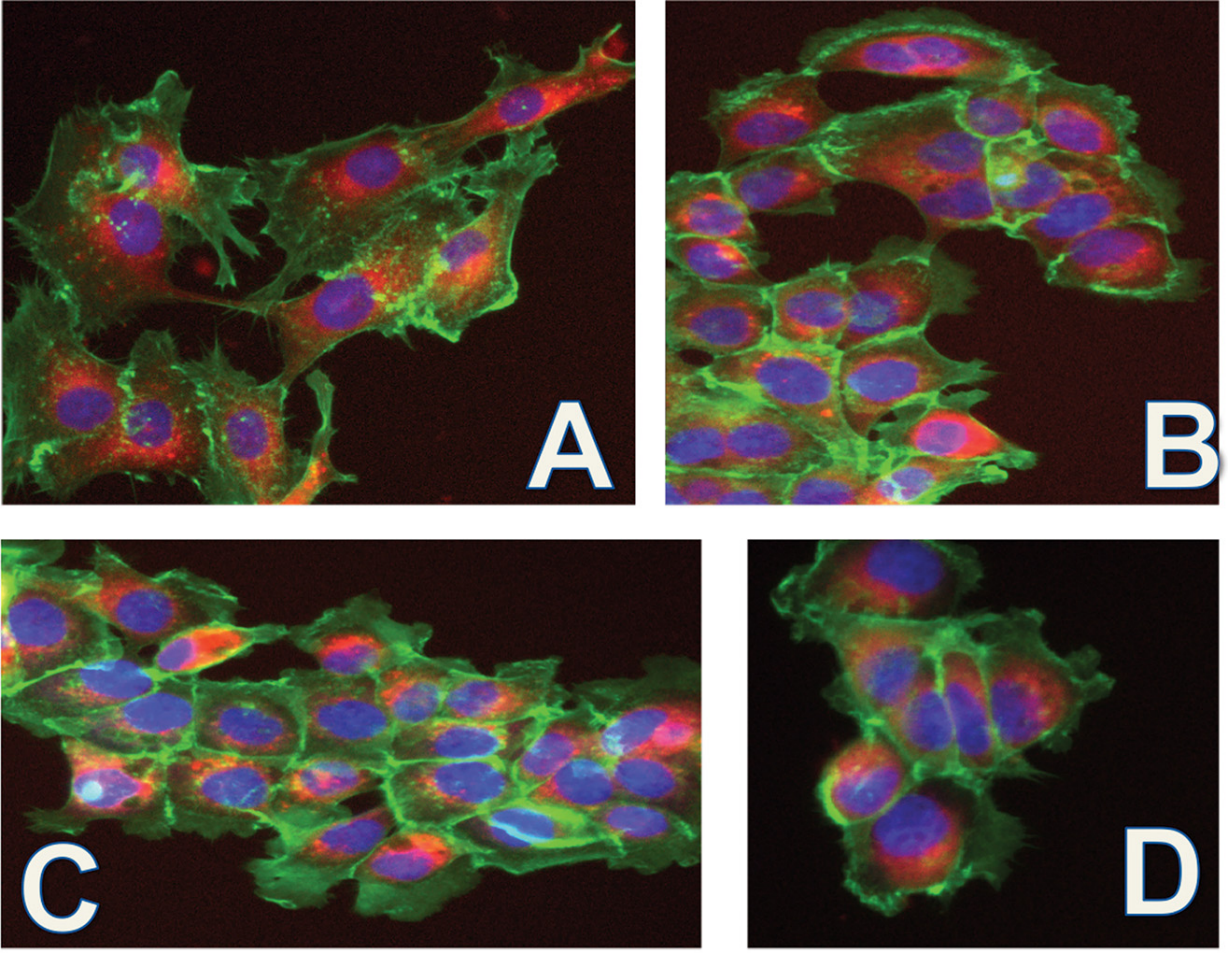
OE33 and SKGT-4 cells stained with compounds 9a-11b (red), nuclear dye Hoechst 33342 (blue), F-actin dye Phalloidin 490 (green): (a) 8a 30 μM, SKGT-4; (b) 9b OE33 40 μM; (c) 10a SKGT 40 μM; (d) 11b OE33 40 μM. All photographs were taken at a magnification of 10×.

Compounds **8a-11b** underwent biological testing to ascertain their localization and cytotoxic properties using esophageal carcinoma OE33, esophagus adenocarcinoma, and well-differentiated SKGT-4 human cell lines (Fig 4). All compounds were successfully taken up into the cell and in accordance with previous research conducted with the same PS core, appear to localize in the endoplasmic reticulum (ER) and Golgi apparatus. The compounds were then subjected to a colorimetric MTS assay to assess their cytotoxicity. The compounds were tested over a range of concentrations with an illumination period of 2 min [37]. Unfortunately, none of the conjugates exhibited any phototoxic effect, which may be due to the relatively short illumination period. As per the graph in Fig 3, *m*-THPC has a considerably large yield of ^1^O_2_ after 2 min of illumination, whereas the conjugates tested only begin to become comparable to *m*-THPC after an illumination period of 12 min.

## Conclusions

We have successfully synthesized a small library of chlorin conjugated NSAIDs through the controlled functionalization of the chlorin core through a modified Steglich reaction. This methodology proved to be simple, robust and produced mono- and tetra-functionalized conjugates in reasonable yields and few synthetic steps. This new reaction pathway also highlights the ability of *m*-THPC, a molecule historically perceived to be difficult to work with, to undergo standard chemical manipulations without subsequent oxidation to its parent porphyrin derivative. All conjugates successfully entered into the cells and whilst no cytotoxicity was observed, this work has served as an initial entry point into the field of PDT and post-treatment inflammation. We hope to fine-tune the cytotoxicity of these porphyrins by synthesizing a larger library of conjugates using more regulatory approved anti-inflammatories. These will then undergo a thorough assessment of their PDT efficacy over a range of irradiation times as suggested by the ^1^O_2_ production assay data we have obtained above. It is hoped that this approach will further progress the reduction of post-PDT inflammation and tumor regrowth, and therefore improve the overall efficiency of PDT. This library of conjugates could also undergo *in vivo* experiments in mice in conjunction with researchers in the biochemists. Ideally, these potential collaborators would administer one of the conjugates intravenously and through the use of Western-blot analysis, observe reduced levels of the pro-inflammatory molecules associated with this tumor regrowth.

## Materials and Methods

All commercial chemicals used were of analytical grade and were supplied by Sigma Aldrich, Frontier Scientific, Inc. and Tokyo Chemical Industry (TCI) and used without further purification unless otherwise stated. Anhydrous THF and diethyl ether were obtained *via* distillation over sodium/benzophenone and dichloromethane dried was obtained *via* distillation over P_2_O_5_. Cell lines were obtained from ^1^H and ^13^C NMR spectra were recorded on a Bruker dpx 400 (400 MHz for ^1^H NMR; 100.6 MHz for ^13^C NMR), Agilent MR400 (400.13 MHz for ^1^H NMR and 100.61 MHz for ^13^C NMR) and Bruker AV 600 (600 MHz for ^1^H NMR; 150.9 MHz for ^13^C NMR. High-Resolution Mass-Spectrometry (HRMS) experiments were measured on a Waters MALDI Q-TOF Premier in positive and negative mode with DCTB (*trans*-2[3-4-*tert*-butylphenyl)-2-2-methyl-2-propenylidene]malononitril) as the MALDI matrix. Melting points were acquired on a Stuart SMP-10 melting point apparatus and are reported uncorrected. Thin layer chromatography (TLC) was performed on silica gel 60 (fluorescence indicator F254;Merck) pre-coated aluminium sheets. Flash chromatography was carried out using Fluka silica gel 60 (230–400 mesh) and aluminium oxide (neutral, activated with 6.5% H_2_O, Brockmann Grade III). Photophysical measurements were performed using EtOAc, THF, and CH_2_Cl_2_ as solvents with UV-vis absorption measurements performed with a Shimadzu Multispec-1501 Spectrometer.

The human esophageal squamous cell carcinoma cell line OE21 and the human adenocarcinoma cell lines SKGT-4 and OE33, derived from Barrett’s esophagus, were purchased from the European Collection of Cell Cultures (ECACC) [38,39].

### Biological Testing

General procedure for cell cultures and the (3-(4,5-dimethylthiazol-2-yl)-5-(3-carboxymethoxyphenyl)-2-(4-sulfophenyl)-2H-tetrazolium) (MTS) cell proliferation assay: Cell lines were seeded at a concentration of 8 × 10^4^ cells per mL into sterile 96-well plates, left to attach overnight and treated. To previously prepared 96-well assay plates containing cells in 100 μL of culture medium, the test compounds at different concentrations and appropriate controls were added. After incubation for 24 h the medium was removed and changed for fresh one, dark controls were left in the dark for next 24 h. To assess the phototoxicity, the rest of the plates were illuminated for 2 min and incubated for 24 h. Finally, 20 μL of MTS dye solution was added to each well of the dark controls and illuminated plates and these were incubated for 3 h and the absorbance was recorded at 470 nm using a 96-well plate reader. Cell lines were seeded at a concentration of 3 × 10^4^ cells per mL into sterile 96-well plates leaving them for 24 h to attach. For imaging experiments, the cell culture medium was removed, replaced with freshly prepared solutions of the porphyrins **8**–**11** of various concentrations in the medium and incubated at 37°C under 5% CO_2_ for 24 h. After that the medium was removed and the cells were fixed with 4% paraformaldehyde (PFA) in medium and then washed with phosphate-buffered saline (PBS). Fluorescent images were collected and analyzed by high content screening and imaging technique (IN Cell 1000 instrument, GE Healthcare). Full details on the cell cultures and treatment, cell proliferation assay, high content imaging and analysis, statistical treatment, and illumination protocol are as given in Vaz *et al*. [37].

### General Synthetic Procedure A

Monofunctionalization of *m-*THPC: EDC (2 eq.), HOBt (2 eq.), K_2_CO_3_ (2 eq.) were added to a 50 mL round bottomed flask and charged with DMF (10 mL). The appropriate anti-inflammatory agent (2–3 eq.) was added and the reaction was allowed to stir for 60 minutes under argon. *m-*THPC (1 eq.) was added to the mixture and the reaction was monitored by TLC and once significant conversion to the mono-substituted species was achieved, the reaction was terminated through the addition of CH_2_Cl_2_ (50 mL). The crude reaction mixture was washed with distilled water (1×30 mL), sat. aq. NaHCO_3_ (1×30 mL), brine (1×30 mL), and distilled water (2×30 mL). The organic phase was dried over anhydrous sodium sulfate, filtered and evaporated under reduced pressure.

### General Synthetic Procedure B

Tetrafunctionalization of *m-*THPC: *m-*THPC (1 eq.) was added to a round-bottomed flask (50 mL) and charged with DMF (10 mL) and K_2_CO_3_ (5–20 eq.). The reaction was left to stir for 60 minutes under argon before the appropriate functionalization reagent (10–20 eq.) was added. The reaction mixture was monitored by TLC and once full conversion to the desired degree of substitution was observed the reaction was terminated through the addition of CH_2_Cl_2_ (50 mL). The work-up proceeds as per general procedure A.

### 5,10,15-Tris(3-hydroxyphenyl)-20-[3-phenyl(2-amino-5-hydroxybenzoate]chlorin / 5,15,20-Tris(3-hydroxyphenyl)-10-[3-phenyl(2-amino-5-hydroxybenzoate]chlorin (8a)

The compound was synthesized using general procedure A: a 50 mL round bottom flask containing 10 mL of DMF, **1** (100 mg, 0.147 mmol), EDC (46.0 mg, 0.294 mmol), HOBt (39.7 mg, 0.294 mmol), K_2_CO_3_ (40.6 mg, 0.294 mmol) and 5-hydroxyanthranilic acid (44.9 mg, 0.297 mmol) was allowed stir for 4 h. NMR indicates a mixture of regioisomers. Column chromatography on silica gel using CH_2_Cl_2_/*n*-hexane/MeOH (3:1:0.2, v/v/v) produced a mixture of monofunctionalized regioisomers in a yield of 43.1 mg of purple crystals (0.053 mmol, 36%). Analytical data: m.p. >300°C; *R*_*f*_ = 0.43 [CH_2_Cl_2_/*n*-hexane/MeOH (3:1:0.2)]; ^1^H NMR (400 MHz, [(CD_3_)_2_SO], 25°C): *δ*_H_ = -1.68 (s, 1H,-N*H*), -1.61 (s, 1H,-N*H*), 4.14 (s, 4H, *H*_β_), 6.84 (m, 2H, Ar-*H*), 7.14 (m, 1H, Ar-*H*), 7.24 (m, 9H, Ar-*H*), 7.47 (m, 7H, Ar-*H*), 8.22 (d, ^3^J_H-H_ = 4.93 Hz, 2H, *H*_β_), 8.3 (s, 2H, *H*_β_), 8.61 (d, ^3^J_H-H_ = 4.93 Hz, 2H, *H*_β_), 9.24 (s, 1H, O*H*), 9.77 (app d, 3H, O*H*) ppm; ^13^C NMR (100 MHz, [(CD_3_)_2_SO], 25°C): *δ* = 36.1, 115.1, 115.5, 119.7, 120.4, 122.3, 123.5, 123.7, 126.2, 128.3, 129.2, 131.7, 139.8, 141.5, 142.9, 143.3, 156.9, 157.2, 164.2 ppm; UV/Vis (EtOAc): *λ*_max_ (lg *ε*) = 419 (6.78), 519 (5.67), 545 (5.48), 598 (5.28), 651 nm (6.03); HRMS (MALDI) calcd for [M]^+^ C_51_H_37_N_5_O_6_ 815.2744, found 815.2731.

### 5,10,15,20-Tetrakis-[3-phenyl(2-amino-5-hydroxybenzoate)]chlorin (8b)

The compound was synthesized using general procedure B: a 50 mL round bottom flask containing 10 mL of DMF, **1** (100 mg, 0.147 mmol), EDC (273 mg, 1.76 mmol), HOBt (238 mg, 1.76 mmol), K_2_CO_3_ (243 mg, 1.76 mmol) and anthranilic acid (269 mg, 1.76 mmol) was allowed stir for 20 h. Recrystallization from ethyl acetate:n-hexane yielded 109 mg of purple crystals (0.089 mmol, 61% yield). m.p. > 300°C; *R*_*f*_ = 0.91 [CH_2_Cl_2_/*n*-hexane/MeOH (3:1:0.2)]; ^1^H NMR (400 MHz, CDCl_3_, 25°C): *δ* = -1.54 (s, br, 2H,-N*H*), 4.16 (s, 4H, *H*_β_)_,_ 7.05 (m, 4H, Ar-*H*), 7.14 (m, 4H, Ar-*H*), 7.22 (m, 8H, Ar-*H*), 7.29 (m, 8H, Ar-*H*) 7.42 (m, 4H, Ar-*H*), 7.55 (m, 4H, Ar-*H*), 8.59 (d, ^3^J_H-H_ = 4.38 Hz, 2H, *H*_β_), 8.63 (d, ^3^J_H-H_ = 4.38 Hz, 2H, *H*_β_) ppm; ^13^C NMR (100 MHz, CDCl_3_, 25°C): *δ* = 35.9, 114.3, 115.0, 115.4, 117.9, 119.6, 120.9, 121.3, 122.8, 123.2, 123.5, 126.3, 128. 2, 128.9, 129.1, 139.7, 139.9, 141.7, 142.3, 156.6, 157.1, 168.2 ppm; UV/Vis (EtOAc): *λ*_max_ (lg *ε*) = 420 (6.62), 517 (5.33), 545 (5.13), 598 (5.28), 651 nm (5.89); HRMS (MALDI) calcd for [M]^+^ C_72_H_52_N_8_O_12_ 1220.3705, found 1220.3693.

### 5,10,15-Tris(3-hydroxyphenyl)-20-[3-(2-{4-*iso*-butylphenyl}propanoate)phenyl]chlorin / 5,15,20-Tris(3-hydroxyphenyl)-10-[3-(2-{4-*iso*-butylphenyl}propanoate)phenyl]chlorin (9a)

The compound was synthesized using general procedure A: a 50 mL round bottom flask containing 10 mL of DMF, **1** (100 mg, 0.147 mmol), EDC (46.0 mg, 0.294 mmol), HOBt (39.7 mg, 0.294 mmol), K_2_CO_3_ (40.6 mg, 0.294 mmol) and ibuprofen (60.6 mg, 0.297 mmol) was allowed stir for 4 h. NMR indicates a mixture of regioisomers. Column chromatography on silica gel using CH_2_Cl_2_/*n*-hexane/MeOH (3:1:0.2, v/v/v) produced a mixture of monofunctionalized regioisomers in a yield of 45 mg of purple crystals (0.05 mmol, 35%). Analytical data: m.p. >300°C; *R*_*f*_ = 0.37 [CH_2_Cl_2_/*n*-hexane/MeOH (3:1:0.2)]; ^1^H NMR (400 MHz, [(CD_3_)_2_SO], 25°C): *δ*_H_ = -1.67 (s, 2 H.-N*H*), 1.12 (m, 9H,-C*H*_3),_ 2.04 (s, 2 H,-C*H*_2_), 3.00 (m, 2 H,-C*H*), 4.13 (s, 4H, *H*_β_), 7.07 (m, 2 H, Ar-*H*), 7.13 (m, 2 H, Ar-*H*), 7.28 (m, 6H, Ar-*H*), 7.46 (m, 10H, Ar-*H*), 8.21 (d, ^3^J_H-H_ = 4.86 Hz, 2H, *H*_β_) 8.35 (s, 2H, *H*_β_), 8.60 (d, ^3^J_H-H_ = 4.86 Hz, 2H, *H*_β_), 9.75 (s, 3H,-O*H*) ppm; ^13^C NMR (100 MHz, [(CD_3_)_2_SO], 25°C): *δ* = 8.1, 9.0, 31.1, 35.8, 46.0, 55.3, 112.5, 114.1, 115.1, 115.3, 117.8, 119.7, 121.5, 122.4, 123.4, 123.9, 125.7, 128.3, 128.5, 129.6, 129.9, 132.2, 132.2, 134.6, 140.2, 142.9, 143.8, 151.9, 157.4, 168.0 ppm; UV/Vis (EtOAc): *λ*_max_ (lg *ε*) = 420 (6.68), 519 (5.54), 545 (5.34), 597 (5.17), 651 nm (5.92); HRMS (MALDI) calcd for [M]^+^ C_57_H_48_N_4_O_5_ 868.3625, found 868.3613.

### 5,10,15,20-Tetrakis-[3-phenyl(2-{4-*iso*-butylphenyl}propanoate)]chlorin (9b)

This target was synthesized using general procedure B: a 50 mL round bottom flask containing 10 mL of DMF, **1** (100 mg, 0.147 mmol), EDC (273 mg, 1.76 mmol), HOBt (238 mg, 1.76 mmol), K_2_CO_3_ (243 mg, 1.76 mmol) and ibuprofen (363 mg, 1.76 mmol) was allowed stir for 18 h. Recrystallization from ethyl acetate:n-hexane yielded 140 mg of purple crystals (0.096 mmol, 65% yield). m.p. >300°C; *R*_*f*_ = 0.91 [CH_2_Cl_2_/*n*-hexane/MeOH (3:1:0.2)]; ^1^H NMR (400 MHz, CDCl_3_, 25°C): *δ*_H_ = -1.57 (s, 2 H.-N*H*), 0.81 (m, 36H,-C*H*_3),_ 1.25 (s, 8 H,-C*H*_2_), 2.37 (m, 8 H,-C*H*), 4.12 (s, 4H, *H*_β_), 7.07 (m, 8 H, Ar-*H*), 7.28 (m, 8 H, Ar-*H*), 7.28 (m, 6H, Ar-*H*), 7.46 (m, 10H, Ar-*H*), 8.21 (d, ^3^J_H-H_ = 4.86 Hz, 2H, *H*_β_) 8.35 (s, 2H, *H*_β_), 8.60 (d, ^3^J_H-H_ = 4.86 Hz, 2H, *H*_β_) ppm; ^13^C NMR (100 MHz, CDCl_3_, 25°C): *δ* = 19.0, 22.1, 30.3, 44.7, 114.6, 114.7, 114.8, 119.3, 119.4, 120.3, 123.3, 123.4, 123.7, 127.6, 129.6, 137.8, 140.4, 144.6 176.2 ppm; UV/Vis (EtOAc): *λ*_max_ (lg *ε*) = 419 (6.71), 518 (5.58), 545 (5.38), 597 (5.16), 651 nm (5.96); HRMS (MALDI) calcd for [M]^+^ C_96_H_96_N_4_O_8_ 1432.7228, found 1432.7215.

### 5,10,15-Tris(3-hydroxyphenyl)-20-[3-phenyl(2-(phenylamino)benzoate]chlorin / 5,15,20-Tris(3-hydroxyphenyl)-10-[3-phenyl(2-(phenylamino)benzoate]chlorin (10a)

The compound was synthesized using general procedure A: a 50 mL round bottom flask containing 10 mL of DMF, **1** (100 mg, 0.147 mmol), EDC (46.0 mg, 0.294 mmol), HOBt (39.7 mg, 0.294 mmol), K_2_CO_3_ (40.6 mg, 0.294 mmol) and fenamic acid (62.6 mg, 0.297 mmol) was allowed stir for 3 h. NMR indicates a mixture of regioisomers. Column chromatography on silica gel using CH_2_Cl_2_/*n*-hexane/MeOH (3:1:0.2, v/v/v) produced a mixture of monofunctionalized regioisomers in a yield of 50.6 mg of purple crystals (0.064 mmol, 45%). Analytical data: m.p. >300°C; *R*_*f*_ = 0.43 [CH_2_Cl_2_/*n*-hexane/MeOH (3:1:0.2)]; ^1^H NMR (400 MHz, [(CD_3_)_2_SO], 25°C): *δ*_H_ = -1.63 (s, 2H,-N*H*), 4.13 (s, 4H, *H*_β_), 6.86 (m, 2H, Ar-*H*), 7.06 (m, 3H, Ar-*H*), 7.23 (m, 8H, Ar-*H*), 7.31 (m, 3H, Ar-*H*), 7.46 (m, 8H, Ar-*H*), 7.67 (s, 1H, Ar-*H*), 8.21 (d, ^3^J_H-H_ = 4.93 Hz, 2H, *H*_β_), 8.61 (m, 2H, *H*_β_), 8.85 (d, ^3^J_H-H_ = 4.93 Hz, 2H, *H*_β_), 9.70 (s, 2H,-O*H*), 9.86 (s, 1H,-O*H*) ppm; ^13^C NMR (100 MHz, [(CD_3_)_2_SO], 25°C): *δ* = 36.1, 114.7, 118.3, 119.7, 121.5, 122.2, 122.3, 122.6, 123.9, 125.4, 128.3, 128.5, 129.9, 131.9, 132.0, 132.4, 134.5, 134.6, 142.8, 143.8, 149.5, 152.0, 156.3, 167.5 ppm; UV/Vis (EtOAc): *λ*_max_ (lg *ε*) = 420 (6.11), 519 (5.69), 544 (5.50), 597 (5.30), 645 nm (6.07); HRMS (MALDI) calcd for [M]^+^ C_57_H_41_N_5_O_5_ 875.3108, found 875.3117.

### 5,10,15,20-Tetrakis[3-phenyl(2-(phenylamino)benzoate]chlorin (10b)

The compound was synthesized using general procedure B: a 50 mL round bottom flask containing 10 mL of DMF, **1** (100 mg, 0.147 mmol), EDC (273 mg, 1.76 mmol), HOBt (238 mg, 1.76 mmol), K_2_CO_3_ (243 mg, 1.76 mmol) and fenamic acid (257 mg, 1.76 mmol) was allowed stir for 20 h. Recrystallization from ethyl acetate:n-hexane yielded 178 mg of purple crystals (0.122 mmol, 83% yield). m.p. >300°C; *R*_*f*_ = 0.91 [CH_2_Cl_2_/*n*-hexane/MeOH (3:1:0.2)] ^1^H NMR (400 MHz, CDCl_3_, 25°C): *δ* = -1.65 (s, br, 2H,-N*H*), 4.17 (s, 4H, *H*_β_), 7.24 (m, 5H, Ar-*H*)_,_ 7.47 (m, 8H, Ar-*H*), 7.62 (m, 8H, Ar-*H*), 7.74 (m, 4H, Ar-*H*) 7.82 (m, 8H, Ar-*H*), 8.04 (m, 4H, Ar-*H*), 8.37 (m, 3H, Ar-*H*), 8.5 (m, 4H, Ar-*H*), 8.64 (m, 4H, Ar-*H*), 8.85 (m, 4H, Ar-*H*), 9.30 (s, 6H, *H*_β_), 9.75 (s, br, 4H,-N*H*) ppm; ^13^C NMR (100 MHz, CDCl_3_, 25°C): *δ* = 36.4, 112.6, 114.6, 114.8, 114.9, 115.5, 118.6, 121.5, 122.2, 122.3, 122.5, 129.1, 129.5, 129.6, 129.7, 129.9, 130.5, 151.9, 156.4, 157.6, 158.5, 167.9 ppm. UV/Vis (EtOAc): *λ*_max_ (lg *ε*) = 420 (6.90), 518 (5.78), 545 (5.59), 598 (5.39), 651 nm (6.16); HRMS (MALDI) calcd for [M]^+^ C_96_H_68_N_8_O_8_ 1460.5160, found 1460.5149.

### 5,10,15-Tris(3-hydroxyphenyl)-20-[3-phenyl(nicotinate]chlorin / 5,15,20-Tris(3-hydroxyphenyl)-10-[3-phenyl(nicotinate]chlorin (11a)

The compound was synthesized using general procedure B: a 50 mL round bottom flask containing 10 mL of DMF, **1** (100 mg, 0.147 mmol), EDC (46.0 mg, 0.294 mmol), Pyridine (0.3 mL) and nicotinic acid (36.2 mg, 0.297 mmol) was allowed stir for 2 h. NMR indicates a mixture of regioisomers. Column chromatography on silica gel using CH_2_Cl_2_/*n*-hexane/MeOH (3:1:0.2, v/v/v) produced a mixture of monofunctionalized regioisomers in a yield of 49.6 mg of purple crystals (0.063 mmol, 43%). Analytical data: m.p. >300°C; *R*_*f*_ = 0.43 [CH_2_Cl_2_/*n*-hexane/MeOH (3:1:0.2)]; ^1^H NMR (400 MHz, [(CD_3_)_2_SO], 25°C): *δ*_H_ = -1.67 (s, 2H,-N*H*), 4.13 (s, 4H, *H*_β_), 7.06 (d, 2H, ^3^J_H–H_ = 8.33 Hz, Ar-*H*), 7.14 (d, 2H, ^3^J_H–H_ = 8.33 Hz, Ar-*H*), 7.25 (m, 8H, Ar-*H*), 7.66 (m, 8H, Ar-*H*), 8.21 (d, 3H, ^3^J_H–H_ = 4.82 Hz, *H*_β_), 8.63 (d, 3H, ^3^J_H–H_ = 4.82 Hz, *H*_β_), 9.21 (s, br, 3H,-O*H*) ppm; ^13^C NMR (100 MHz, [(CD_3_)_2_SO], 25°C): *δ* = 36.7, 115.5, 120.4, 122.3, 126.2, 128.3, 129.2, 131.7, 139.8, 141.5, 142.9, 143.3, 156.9, 157.2, 165.4 ppm; UV/Vis (EtOAc): *λ*_max_ (lg *ε*) = 420 (6.44), 507 (5.51), 534 (5.31), 589 (5.01), 645 nm (5.94); HRMS (MALDI) calcd for [M]^+^ C_50_H_35_N_5_O_5_ 785.2638, found 785.2615.

### 5,10,15,20-Tetrakis[3-phenyl(nicotinate]chlorin (11b)

The compound was synthesized using general procedure B: a 50 mL round bottom flask containing 10 mL of DMF, **1** (100 mg, 0.147 mmol), EDC (273 mg, 1.76 mmol), pyridine (1 mL) and nicotinic acid (216 mg, 1.76 mmol) was allowed stir for 20 h. Recrystallization from ethyl acetate:n-hexane yielded 113 mg of purple crystals (0.102 mmol, 70% yield). m.p. >300°C; *R*_*f*_ = 0.91 [CH_2_Cl_2_/*n*-hexane/MeOH (3:1:0.2)]; ^1^H NMR (400 MHz, CDCl_3_, 25°C): *δ*_H_ = -1.67 (s, br, 2H,-N*H*), 4.17 (s, 4H, *H*_β_), 7.60 (m, 8H, Ar-*H*_chlorin),_ 7.83 (m, 8H, Ar-*H*_chlorin)_, 8.04 (m, 4H, Ar-*H*_Nico_), 8.49 (m, 4H, Ar-*H*_Nico_), 8.85 (m, 8H, Ar-*H*_Nico)_ 9.29 (s, br, 6H, *H*_β_) ppm; ^13^C NMR (100 MHz, CDCl_3_, 25°C): *δ* = 114.51, 119.52, 121.27, 127.46, 128.41, 155.85, 171.01 ppm; UV/Vis (EtOAc): *λ*_max_ (lg *ε*) = 420 (6.44), 507 (5.51), 534 (5.31), 589 (5.01), 645 nm (5.94); HRMS (MALDI) calcd for [M]^+^ C_68_H_44_N_8_O_8_ 1100.3282, found 1100.3292.
